# The role of long-term target and masker talker familiarity in children’s speech-in-speech recognition

**DOI:** 10.3389/fpsyg.2024.1369195

**Published:** 2024-05-09

**Authors:** Mary M. Flaherty

**Affiliations:** Department of Speech and Hearing Science, University of Illinois at Urbana-Champaign, Champaign, IL, United States

**Keywords:** sentence recognition, familiarity, voice, children, speech-in-speech

## Abstract

**Objectives:**

This study investigated the influence of long-term talker familiarity on speech-in-speech recognition in school-age children, with a specific emphasis on the role of familiarity with the mother’s voice as either the target or masker speech.

**Design:**

Open-set sentence recognition was measured adaptively in a two-talker masker. Target and masker sentences were recorded by the adult mothers of the child participants. Each child heard sentences spoken by three adult female voices during testing; their own mother’s voice (familiar voice) and two unfamiliar adult female voices.

**Study sample:**

Twenty-four school age children (8–13 years) with normal hearing.

**Results:**

When the target speech was spoken by a familiar talker (the mother), speech recognition was significantly better compared to when the target was unfamiliar. When the masker was spoken by the familiar talker, there was no difference in performance relative to the unfamiliar masker condition. Across all conditions, younger children required a more favorable signal-to-noise ratio than older children.

**Conclusion:**

Implicit long-term familiarity with a talker consistently improves children’s speech-in-speech recognition across the age range tested, specifically when the target talker is familiar. However, performance remains unaffected by masker talker familiarity. Additionally, while target familiarity is advantageous, it does not entirely eliminate children’s increased susceptibility to competing speech.

## 1 Introduction

The presence of background noise, especially competing talkers, presents an especially challenging communication environment for children. This is because children are more vulnerable to interference from background speech compared to adults, making speech understanding more difficult in multitalker contexts well into adolescence (Hall et al., 2002; Leibold and Buss, 2013). When recognizing speech in the presence of one or more competing talkers, children require a more advantageous signal-to-noise ratio (SNR) to achieve the same level of performance as adults until they reach around 13 years of age or older (Leibold and Buss, 2013; Corbin et al., 2016; Leibold et al., 2016; Buss et al., 2017; Flaherty et al., 2019). Despite having potentially negative consequences for children’s language development and communication, this prolonged developmental trajectory for speech-in-speech recognition is not well understood. New findings indicate that it stems partially from children’s immature sound segregation and selective attention, along with their restricted capacity to utilize sparse spectro-temporal cues during speech recognition (Buss et al., 2017, 2019).

One factor influencing speech recognition in both children and adults is the familiarity with the target voice, known as talker familiarity. Talker familiarity has been shown to enhance spoken word recognition in both quiet and noisy conditions for children (Levi, 2015; Flaherty et al., 2024) and in the presence of a single competing talker for both infants and adults (Barker and Newman, 2004; Johnsrude et al., 2013). Knowledge of vocal characteristics of a talker appear to enhance adult listeners ability to attend to target speech and ignore background sounds, suggesting a potential benefit for children in these contexts. For adults, talker familiarity not only improves word and sentence recognition in noise, but also improves recognition memory and decreases processing time (Van Lancker et al., 1985; Clarke and Garrett, 2004; Theodore et al., 2015). However, these processes in children remain relatively unexplored, with existing studies largely concentrating on talker voice training scenarios (Levi, 2015; Levi et al., 2019). Additionally, there are no studies, to our knowledge, that have explored talker familiarity effects on school-age children’ speech-in-speech recognition, focusing instead on target talker familiarity effects in non-speech noise. Given that children frequently acquire speech and language skills through interactions with familiar voices in multi-speaker environments, it becomes imperative to determine the extent to which talker familiarity influences their performance in these contexts.

Studies investigating the impact of talker familiarity in school-age children reveal that short-term familiarity with a specific talker can significantly enhance speech-in-noise recognition. Children between the ages of 7 and 12, explicitly trained to identify voices over a 5-day period, exhibit improved word recognition in noise when presented by a familiar talker compared to an unfamiliar one (Levi, 2015). However, this focus on explicit short-term familiarity does not address the influence of implicit, or passive talker familiarity acquired over time. Research in adults indicates that explicitly-induced familiarity effects (Nygaard et al., 1994) tend to be smaller in magnitude compared to implicit familiarity effects (Kreitewolf et al., 2017). A recent study exploring implicit short-term familiarity observed that children aged 8–12, exposed passively to a specific voice through a computer game over 5 days, demonstrated enhanced word recognition when that voice was presented in classroom noise (Flaherty et al., 2023). This suggests that implicitly acquired familiarity can indeed influence speech recognition in noise for children. Barker and Newman (2004) were able to show the usefulness of implicit long-term familiarity in 6–8 month-old infants by testing their recognition of words spoken by their mother in the presence of a competing unfamiliar female voice, utilizing a preferential listening paradigm. Despite the perceptual similarity between the competing voices, infants adeptly employed talker familiarity as a cue to segregate the competing female talkers, thereby facilitating their speech-in-speech recognition. This underscores the substantial impact of naturally-acquired long-term familiarity and its pivotal role in shaping the auditory processing abilities of children from infancy.

Beyond Barker and Newman (2004) investigation, other investigations of long- or short-term talker familiarity effects on speech-in-speech recognition are limited to research in adults. Though children appear to use talker familiarity in competing noise maskers, the extent to which familiarity cues are beneficial to children in multitalker contexts has not been established. The nature of the interfering sounds significantly affects children’s ability to recognize speech and is typically categorized into two forms of masking, each having different developmental trajectories. Energetic masking arises from the physical overlap between target and masker sounds, rendering the peripheral auditory system unable to represent them as distinct sources. In contrast, informational masking occurs when the listener can hear both the target and masker speech but struggles to distinguish and process them separately due to the complexity of the auditory scene. Children’s ability to recognize speech in a noise masker typically matures by 8 to 11 years of age, whereas recognition in a speech masker does not mature until the teenage years (Leibold and Buss, 2013; Corbin et al., 2016; Buss et al., 2017). Therefore, the type of masker is also expected to impact the processes involved in and the extent to which children recognize the speech spoken by a familiar talker. Given their greater difficulty recognizing speech in a speech masker, talker familiarity may be especially important for children in these contexts.

By only focusing on talker familiarity effects in children within the context of noise maskers, prior studies have not investigated how masker familiarity might influence children’s performance. Given that talker familiarity could serve as a segregation cue that can improve speech-in-speech recognition, the familiarity with the masker speech could also play an important role in these processes. Familiarity with a masker voice has the potential to facilitate children’s speech recognition by improving segregation or selective attention. Repeated exposure to a voice may allow the auditory system to become more efficient at recognizing and processing that specific voice, making it easier to group the target from the masker speech. This faciliatory effect could also come from a reduction in cognitive load or listening effort, freeing up resources to complete other tasks, including selective attention to the target speech. Alternatively, masker familiarity could potentially have a detrimental impact on target speech recognition by diverting the child’s attention away from the target speech, especially if the masker speech is spoken by their own mother. It is unknown how effectively school-age children are able to inhibit a familiar voice, or the voice of their own mother, in order to attend to target speech. However, research suggests that children exhibit poorer selective attention abilities (Coch et al., 2005; Karns et al., 2015), indicating they may face greater challenges in ignoring a familiar masker voice compared to adults.

Investigations of the impact of masker talker familiarity in adults have found mixed results. Using a version of the coordinate response measure (CRM), Johnsrude et al. (2013) demonstrated that both younger and older adults’ word recognition improved when the target or masker talker was a highly familiar voice, in this case the participant’s long-term spouse. That is, not only were adults better recognizing words spoken by their spouse in the presence of a competing unfamiliar voice of the same gender, but they were also better at ignoring their spouse’s voice when it was the competing masker speech. Their results were interpreted as evidence that talker familiarity functions as a knowledge-based segregation cue, as opposed to operating solely as a template matching mechanism or reflecting a tendency to selectively focus on a familiar voice. However, more recent investigations have found conflicting results, with adults not exhibiting a speech recognition benefit related to masker talker familiarity (Domingo et al., 2020). The adults tested displayed no distinction in their ability to recognize speech whether the masking voice was a familiar one or when both the intended speech and the masking were from unfamiliar voices. In fact, there was a non-significant trend toward a detrimental effect of masker familiarity, suggesting a tendency to incorrectly attend to the familiar masker voice. Reasons for the disparities in studies were explained as an increased memory load due to the task being more challenging than the CRM task in the earlier study. Consequently, the role or potential benefit of masker familiarity is not well understood in any age group.

The purpose of the current study is to investigate the influence of long-term target and masker talker familiarity on children’s speech-in-speech recognition. To achieve this, the child’s own mother’s voice was used as either target or masker speech, leveraging the inherent long-term familiarity with their mother. Children’s open-set sentence recognition was measured adaptively in a two-talker female masker using three experimental conditions: (1) familiar target/unfamiliar masker; (2) familiar masker/unfamiliar target; and (3) unfamiliar target/unfamiliar masker. Condition 1 was designed to examine effects of long-term target familiarity on speech-in-speech recognition and to determine whether such familiarity would impact age effects typically associated with children’s performance. Condition 2 aimed to explore whether masker talker familiarity would affect recognition performance, given our limited understanding of familiar masker effects, and its potential to shed light on the mechanisms underlying the familiarity benefit. Condition 3 served as a baseline comparison condition, in which performance was expected to be the poorest.

## 2 Participants and methods

### 2.1 Participants

Listeners were 24 children (8.5–13.1 years., M = 10.9). All children were native speakers of American English and had normal hearing, with thresholds of ≤ 20 dB HL for octave frequencies between 250 and 8,000 Hz (American National Standards Institute, 2018). None had a history of cognitive problems or developmental delays, per parent report. The children’s mothers (35.5–51.97 years, M = 42.5) were also recruited to record the speech stimuli. Mother’s provided written informed consent. Children provided written assent. The child’s accompanying parent provided written consent. The study protocol was approved by the institutional review board of the University of Illinois Urbana-Champaign (#19120). All participants were paid for their participation.

### 2.2 Stimuli and conditions

Target and masker stimuli were both composed of Bamford–Kowal–Bench sentences (BKB; Bench et al., 1979), recorded by the mothers of the child participants. All mothers were monolingual, native English speakers, and spoke a Midwestern dialect. These sentences are appropriate for use with children as young as 5 years of age. This corpus includes 21 lists of 16 sentences, each with three to four keywords, for a total of 50 keywords per list. All 21 lists were recorded by each mother in the laboratory prior to their child’s visit. Both target and masker stimuli were recorded at a sampling rate of 44.1 kHz in a sound-treated booth using a condenser microphone (Shure-KSM42) mounted approximately 6 in. from the talker’s mouth. Target stimuli included Lists 1- 18. Mothers read from a tablet controlled by an experimenter outside the booth. The mother was presented with one sentence at a time so that the experimenter could control the pacing of the sentences and could correct for errors. All sentences were trimmed for silence and root-mean-square (RMS) equalized to the same pressure level.

For the masker, a two-talker speech stream was also created from each target talker’s BKB sentences. Lists 19, 20 and 21 were used to form the two masker streams. One masker stream contained List 19 and the first half of List 20. The second half of list 20 and all of list 21 were used for the other, so that the two masker streams were of equal length. The individual masker streams were manually edited to reduce silent pauses longer than 200 ms, resulting in samples that were approximately 45 s in duration. The two masker speech streams were balanced for overall root-mean-square level and mixed. The masker was gated on 1 s before the target sentences and gated off 1 s after the target sentence ended.

### 2.3 Procedure

Children were seated in a sound-treated booth, approximately 1 m in front of and directly facing a single loudspeaker during testing. The target and masker stimuli were mixed digitally and played from a soundcard (Scarlett 2i2, Focusrite), amplified (Applied Research and Technology, SLA-4), and presented through the loudspeaker (JBL-1). Presenting all stimuli from a single loudspeaker eliminated the presence of spatial cues. ß A custom MATLAB script was used to select and present the stimuli. Children were instructed that they would be listening to a single female talker and repeating back what that talker said. They were also told they would hear two other female talkers speaking in the background, but to focus on the target talker and ignore the two people talking in the background. Children were told to guess when not sure. They were told that they may or may not recognize one or more of the voices, but that they should be sure to always repeat the single female talker and not the talker in the background. They were told that the voice they were repeating back would be different in each condition.

Responses were spoken aloud while facing a microphone mounted on the booth wall. The input was routed through the audiometer so that the experimenter could hear the response. The experimenter had a clear view of the child’s face through the booth window during testing. A tester outside the booth scored each keyword as correct or incorrect using the computer interface.

An adaptive procedure was used to estimate speech reception thresholds (SRT). During the procedure, the level of the two-talker masker remained fixed at 70 dB SPL at 1 m, while the level of the target signal was adjusted based on the child’s performance on each trial. The SRT estimates were based on individual responses obtained for two interleaved adaptive tracks. Both tracks followed a one-down, one-up tracking rule, but they differed in the criteria used for counting the sentence as correct. In one track, a single correct keyword was sufficient to decrease the SNR, while in the other track, three or more correct keywords were required. An incorrect response led to an increase in the SNR. Initially the SNR was adjusted using a step size of 4 dB. After the first reversal, the step size was reduced to 2 dB. Each of the two tracks were comprised of 20 target sentences, for a total of 40 target sentences per condition. Word level data from both tracks were combined and fitted with a logit function with asymptotes at 0 and 100% correct. The use of two tracks, each with distinct criteria (a lax and a strict criterion), facilitated the estimation of the psychometric function slope and SRT, thus characterizing performance across both easy and challenging SNRs. The SRT was defined as the SNR associated with 50% correct. Data fits were associated with *r*^2^ values ranging from 0.72 to 0.99, with a median value of 0.88.

There were 16 practice trials prior to testing to familiarize children with the task. Neither the talker nor the sentences used during practice would be heard in the testing phase. During practice, both adaptive tracks started at 10 dB SNR. All children were able to understand the instructions and successfully complete the practice phase. For testing, both adaptive tracks started with a signal level of 7 dB SNR and then were adjusted within each condition as described above.

During testing, each listener heard three voices: their mother’s voice and two unfamiliar voices. The three conditions tested were: (1) familiar target/unfamiliar masker, (2) unfamiliar target/familiar masker, and (3) unfamiliar target/unfamiliar masker, wherein the familiar stimuli were spoken by the listener’s mother and the unfamiliar stimuli were spoken by the unfamiliar females (other child participant’s mothers). Each voice only served once as the target voice and once as masker voice across condition. Specifically, in the familiar target/unfamiliar masker condition, the target sentences were spoken by the mother, and the masker sentences were spoken by one of the unfamiliar talkers (unfamiliar talker#1). In the unfamiliar target/familiar masker condition, the masker sentences were spoken by the mother and the target sentences were spoken by the other unfamiliar talker (unfamiliar talker#2). In the unfamiliar target/unfamiliar masker condition, the target sentences were spoken by the first unfamiliar talker (unfamiliar talker#1), and the masker sentences were spoken by the other unfamiliar talker (unfamiliar talker#2).

After the 16-sentence training block, the three conditions were tested in separate blocks with block order randomized across children. The starting sentence list number was randomized for each child and continued in numerical order of the BKB sentence lists.

### 2.4 Statistical analysis

Statistical analyses were completed with R software (R Core Team, 2019), using the nlme package (Pinheiro et al., 2016). Linear regression was used to examine the effects of age on SRTs. For analyses of age as a continuous variable, a log_10_ transform was applied to age in years based on the rationale that maturation progresses more rapidly for younger children compared to older children (e.g., Buss et al., 2017). Linear mixed-effects models with a random intercept for each subject were used. Follow-up pairwise comparisons used least square mean differences Tukey testing with a significance level of α = 0.05.

## 3 Results

Data from two children were omitted from analysis as their SRTs in at least one condition exceeded 4 standard deviations from the mean. In both instances, the children struggled to ignore the masker, resulting in exceptionally high SRT values surpassing +30 dB SNR. For one child this was for the Familiar Masker condition (SRT = +40 dB SNR); for the other child this was for the Unfamiliar baseline condition (SRT = +31 dB SNR). The final analyses included 22 child participants (mean age = 11.1 years). These mothers’ voices were still used as unfamiliar speech for other children. There were no noted patterns in the data related to these two mothers’ voices.

**FIGURE 1:**
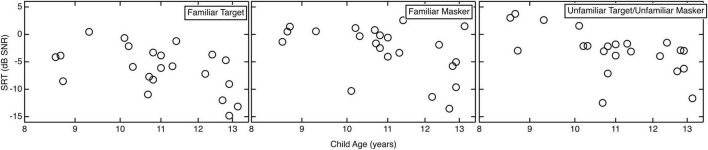
Individual SRTs plotted as a function of child age on a log scale for each of the three conditions.

As seen in Figure 1, SRTs tended to decrease (improve) with increasing age across all conditions. Mean SRTs were −6.2 dB SNR (SD = 4.1) for the familiar target/unfamiliar masker condition, −3.1 dB SNR (SD = 4.1) for unfamiliar target/familiar masker, and −2.9 (SD = 4.6) for the unfamiliar target/unfamiliar masker condition. Performance appeared to be the best in the familiar target condition, in which the target voice was mother’s voice. Performance appeared to be poorer when the masker was familiar, or when both the target and masker were unfamiliar. This suggests a benefit of hearing the mother’s voice as the target voice, but not when the mother’s voice was the masking voice.

A linear mixed model (LMM) was used to examine the statistical significance of these observed trends. The dependent measure was SRT; age and condition as well as their interaction were the fixed effects, and participant was included as a random factor. Age was included as a continuous variable. There was an overall main effect of condition [*F*(2, 40) = 5.96, *p* = 0.0054], a main effect of age [*F*(1, 20) = 18.46, *p* = 0.00035], and no interaction *p* = 0.9596. Model parameter estimates are shown in Table 1. The main effect of age reflects improved performance with increasing age across conditions, as expected. Pairwise comparisons (one-tailed) indicated significantly better SRTs when the target voice was the mothers compared to when both target and masker was unfamiliar (*p* = 0.0052). SRTs were also significantly better in that condition compared to when the mother’s voice was the masking voice (*p* = 0.0053). There was no significant difference in SRTs when the masker was the mother’s voice compared to when both target and masker were unfamiliar (*p* = 0.4902).

**TABLE 1:** Parameter estimates for the mixed effects regression model analyzing SRTs as a function of target/masker familiarity condition and child age on a log scale.

	β	SE	df	*t*	*p*
Intercept	-2.75	0.8008	59.33	-3.43	**0**.**0005**
Age	-16.21	5.94	59.33	-2.73	**0**.**00417**
Familiar target/ unfamiliar masker	-3.36	1.09	40	-3.081	0.00187
Unfamiliar target/ familiar masker	-0.20	1.09	40	-0.186	0.42658
Age × familiar target/unfamiliar masker	1.72	8.08	40	0.213	0.41606
Age × unfamiliar target/familiar masker	-0.48	8.08	40	-0.060	0.47628

β, coefficient estimate, SE, standard error, df, degrees of freedom. Bolded values indicate significance at α = 0.05.

The unfamiliar target/unfamiliar masker condition served as the referent condition.

**FIGURE 2:**
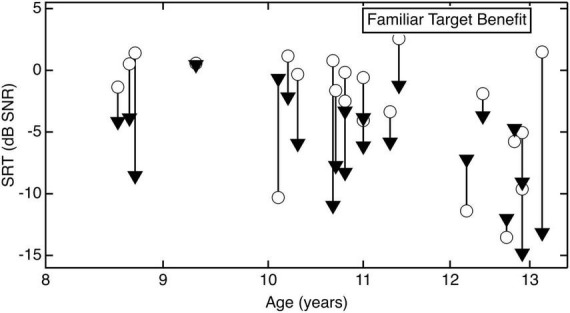
Individual data for each child listener when the target talker was familiar, plotted as a function of age. The circles represent SRTs in the unfamiliar target/unfamiliar masker condition, and the triangles represent SRTs in the familiar target/unfamiliar masker condition. The line connecting the symbols indicates the improvement in performance for each child.

When considering the magnitude of the effect, the absence of an interaction between age and condition demonstrates that the benefit of target familiarity did not depend on child age. For reference, the magnitude of benefit in the familiar target/unfamiliar masker condition for individual children is plotted in Figure 2. The circles represent SRTs in the unfamiliar target/unfamiliar masker condition, and the triangles represent SRTs in the familiar target/unfamiliar masker condition. The line connecting the symbols indicates the improvement in performance for each child.

## 4 Discussion

The current study findings demonstrate that long-term target talker familiarity does facilitate children’s speech-in-speech recognition, at least when that voice is their mother. This was consistent across the age range tested, indicating children between the ages of 8 to 13 years were able to benefit from target familiarity to a similar degree. Masker familiarity, on the other hand, did not consistently impact performance, with children performing similarly when the masker voice was familiar as when it was unfamiliar, though with some variability. Regardless of target or masker familiarity, age effects on speech-in-speech recognition were still observed, with younger children having higher (worse) thresholds relative to older children, even within the 8- to 13-year-old age group. This suggests that though target familiarity is beneficial, it does not entirely reduce children’s increased susceptibility to competing speech.

When first considering the effects of target familiarity on children’s speech-in-speech recognition, the only research to our knowledge to examine this was done in infants using a variant of the headturn preference paradigm (Barker and Newman, 2004). In that study, the target voice was the infant’s mother, which was presented in the context of a one-talker masker consisting of an unfamiliar female voice. Based on listening times, the authors determined that infants could segregate the two competing female voices when the target voice was their own mother, compared to when it was unfamiliar. The current results extend those findings to school-age children using an open-set sentence recognition task with a two-female talker masker, suggesting familiarity can improve recognition of a familiar target talker in the presence of the increased informational masking elicited by two-talker speech.

Prior research in adults examining effects of long-term familiarity with a target talker on speech-in-speech recognition are also consistent with the current findings. Like the children in the present study, adults also show better speech-in-speech recognition when the target talker is their long-term spouse or friend compared to an unfamiliar talker of the same gender (Johnsrude et al., 2013; Domingo et al., 2020). Familiarity with a voice may facilitate target speech recognition by improving segregation, promoting attention to the target voice, or by facilitating the recognition of speech based on fewer cues. However, the precise mechanism is not well understood, particularly when only examining the effects of the familiar target talker.

When considering the effects of masker familiarity, the current study found that children’s speech recognition was not consistently influenced, positively or negatively, by familiarity with the masker voice. Their performance did not differ whether the competing speech was their mother’s voice or another mother’s voice. This lack of a consistent effect raises questions about the underlying mechanisms responsible for familiarity benefits during speech-in-speech recognition, suggesting that the observed benefits of target familiarity may not be solely attributed to improved segregation.

Though this has not previously been studied in children, the few studies of masker familiarity in adults have found mixed results. While Johnsrude et al. (2013) found better closed-set speech recognition when the masker was their long-term spouse compared to an unfamiliar talker of the same gender, subsequent investigations of masker familiarity were unable to replicate these findings (Domingo et al., 2020). While these cited investigations used substantially different tasks (closed-set) and stimuli (single-talker masker) compared to the current study, they all focused on effects of implicit long-term masker familiarity. Other studies have examined familiarity with other qualities of the masker, aside from the talker’s voice, and have found they can impact performance. For example, linguistic familiarity, or familiarity with the language being spoken, can negatively impact speech-in-speech recognition. Garcia and Cooke (2006) found that being familiar with the competing language of the masker impairs performance for adults, leading to poorer consonant recognition compared to when the competing language was not familiar to the listener. Similarly, data from children demonstrate they have better sentence recognition in a two-talker masker when the masking language is not familiar to them (Calandruccio et al., 2016). However, in these studies it is difficult to tease apart whether it is familiarity with the masker language or dissimilarity between the target and masker that leads to these effects.

The absence of a clear masker familiarity benefit in the current study may suggest that target familiarity effects are not primarily due to improved segregation. This is because, if segregation is improved by familiarity, it would be expected to improve performance whether the masker or target was familiar. Instead of improving segregation, familiarity could increase attention to the familiar target voice, encouraging children to prioritize or focus more effectively on the target. Talker familiarity could also facilitate processing of specific acoustic features associated with a familiar voice. Children might be more attuned to the pitch, intonation, or other distinctive characteristics of voices they are familiar with, aiding in speech recognition. Interestingly, in the current study there were some children had difficulty ignoring the masker when it was their mother, and thus experienced a detrimental effect of masker familiarity. This finding was not statistically significant but is in line with Domingo et al. (2020), which showed a similar non-significant negative impact of familiarity with the masker. For some children, ignoring their mother’s voice may be difficult and thus indicative of impaired selective attention related to familiarity. However, future research is needed to explore the role that masker familiarity plays in children’s speech recognition.

An additional factor to consider is the potential impact of differences in talker fundamental frequency (F0) on children’s performance. Though a robust acoustic cue for adults in these contexts, existing research indicates that children do not utilize F0 differences to the same extent as adults, requiring larger F0 differences between competing talkers and benefitting to a lesser degree from these differences (Flaherty et al., 2019, 2021). In accordance with these findings, it would be anticipated that the 8-to 13-year-olds in the current study would experience some degree of benefit from F0 differences, provided those differences exceeded three semitones. However, upon analyzing the mean F0 of each mother and the semitone difference between talkers for each condition, it was found that there were only three instances where the semitone difference between the target/masker talker exceeded three semitones. Consequently, F0 differences between mothers are not expected to have influenced performance significantly. Nonetheless, an exploratory analysis was conducted to investigate the relationship between SRTs and F0 differences (in semitones) between competing talkers for each child within each condition. When controlling for age, a correlation between F0 differences and SRTs was observed only in the condition with the unfamiliar target/unfamiliar masker (*r* = −0.479, *p* = 0.041), but not for the other conditions. Intriguingly, the largest semitone differences were identified in the unfamiliar target/unfamiliar masker condition, which also demonstrated poorer performance relative to the familiar target condition. This suggests that children might have been compelled to rely on F0 differences when listening to unfamiliar talkers. Despite the study’s small sample size and the fact that F0 differences were not the primary focus, these findings indicate that children use acoustic cues beyond F0 when the target talker is highly familiar. However additional research is needed to explore what additional acoustic cues, including indexical information, that may have influenced speech recognition beyond talker familiarity effects.

## 5 Conclusion

In conclusion, this study provides valuable insights into the role of long-term talker familiarity in children’s speech-in-speech recognition. The findings consistently demonstrate that familiarity with the target voice, at least when it is the mother, facilitates speech recognition in the presence of competing speech for children between the ages of 8 to 13 years. This suggests a mitigating effect of talker familiarity on the impact of masker interference on speech recognition in children. These results extend prior research conducted with infants and aligns with similar patterns observed in adults with long-term familiarity with a spouse’s voice. Interestingly, masker familiarity did not consistently impact children’s performance, indicating that the benefits of familiarity may not extend uniformly to all aspects of the listening environment. While some variability was observed, children in the age group tested generally performed similarly when the masker voice was familiar as when it was unfamiliar. The lack of a consistent effect raises questions about the underlying mechanisms responsible for familiarity benefits during speech-in-speech recognition, suggesting that the observed benefits of target familiarity may not be attributed soley to improved segregation. However, future investigations with a larger sample size and wider age range are needed to definitively rule out the role of masker familiarity.

Our study contributes to the existing literature by addressing gaps in research on the effects of long-term target talker familiarity in school-age children using an open-set sentence recognition task. The results emphasize that, while target familiarity is beneficial, it does not entirely eliminate children’s susceptibility to competing speech, as evidenced by age-related differences in speech-in-speech recognition across all conditions. Moreover, the findings hint at the complexity of factors influencing speech recognition in children, such as the potential impact of talker familiarity on children’s ability utilize differences in talker voice characteristics. Future research endeavors could delve deeper into the specific acoustic cues or cognitive processes contributing to the observed familiarity benefits.

The findings of this study hold significant implications for audiologic practice, particularly in the domain of pediatric audiology. The demonstrated influence of long-term target talker familiarity on children’s speech-in-speech recognition underscores the importance of considering individualized communication strategies in pediatric rehabilitation. Audiologists working with school-age children may integrate the knowledge that familiarity, especially with the mother or parental female guardian’s voice, can enhance speech recognition in challenging listening conditions. This highlights the potential value of involving parents and caregivers in therapeutic interventions, encouraging consistent and familiar communication patterns. Moreover, recognizing the age-related differences in speech-in-speech recognition emphasizes the need for tailored approaches, with additional support and attention for younger children who may exhibit higher thresholds in challenging auditory environments. The study’s insights into masker familiarity and the exploration of acoustic cues further encourage audiologists to adopt a nuanced understanding of the factors influencing speech processing in pediatric populations. Ultimately, these findings contribute to the refinement of evidence-based practices in audiology, guiding clinicians in optimizing outcomes for children with hearing challenges.

## Data availability statement

The raw data supporting the conclusions of this article will be made available by the author, without undue reservation.

## Ethics statement

The studies involving humans were approved by the University of Illinois Urbana-Champaign, Office for Protection of Research Subjects. The studies were conducted in accordance with the local legislation and institutional requirements. Written informed consent for participation in this study was provided by the participants’ legal guardians/next of kin.

## Author contributions

MF: Conceptualization, Data curation, Formal analysis, Funding acquisition, Investigation, Methodology, Project administration, Resources, Software, Supervision, Validation, Visualization, Writing – original draft, Writing – review & editing.

## References

[B1] American National Standards Institute (2018). *American national standard specification for audiometers (ANSI S3.6-2018).* Washington, DC: American National Standards Institute.

[B2] Barker B. A. Newman R. S. (2004). Listen to your mother! The role of talker familiarity in infant streaming. *Cognition* 94 45–53. 10.1016/j.cognition.2004.06.001 15582622

[B3] Bench J. Kowal A. Bamford J. (1979). The BKB (Bamford-Kowal-Bench) sentence lists for partiallyhearing children. *Br. J. Audiol*. 13, 108–112. 10.3109/03005367909078884 486816

[B4] Buss E. Hodge S. E. Calandruccio L. Leibold L. J. Grose J. H. (2019). Masked sentence recognition in children, young adults, and older adults: Age-dependent effects of semantic context and masker type. *Ear Hear.* 40 1117–1126. 10.1097/AUD.0000000000000692 30601213 PMC6599542

[B5] Buss E. Leibold L. J. Porter H. Grose J. H. (2017). Speech recognition in one- and two-talker maskers in school-age children and adults: Development of segregation and glimpsing. *J. Acoust. Soc. Am.* 141 2650–2660. 10.1121/1.4979936 28464682 PMC5391283

[B6] Calandruccio L. Leibold L. J. Buss E. (2016). Linguistic masking release in school-age children and adults. *Am. J. Audiol.* 25 34–40. 10.1044/2015 26974870 PMC4832874

[B7] Clarke C. M. Garrett M. F. (2004). Rapid adaptation to foreign-accented English. *J. Acoust. Soc. Am.* 116 3647–3658. 10.1121/1.1815131 15658715

[B8] Coch D. Sanders L. D. Neville H. J. (2005). An event-related potential study of selective auditory attention in children and adults. *J. Cogn. Neurosci.* 17 605–622. 10.1162/0898929053467631 15829081

[B9] Corbin N. E. Bonino A. Y. Buss E. Leibold L. J. (2016). Development of open-set word recognition in children: Speech-shaped noise and two-talker speech maskers. *Ear Hear.* 37 55–63. 10.1097/AUD.0000000000000201 26226605 PMC4684436

[B10] Domingo Y. Holmes E. Johnsrude I. S. (2020). The benefit to speech intelligibility of hearing a familiar voice. *J. Exp. Psychol. Appl.* 26 236–247. 10.1037/xap0000247 31524431

[B11] Flaherty M. M. Buss E. Leibold L. J. (2019). Developmental effects in children’s ability to benefit from F0 differences between target and masker speech. *Ear Hear.* 40 927–937. 10.1097/aud.0000000000000673 30334835 PMC6467703

[B12] Flaherty M. M. Buss E. Leibold L. J. (2021). Independent and combined effects of fundamental frequency and vocal tract length differences for school-age children’s sentence recognition in a two-talker masker. *J. Speech Langu. Hear. Res.* 64 206–217. 10.1044/2020_JSLHR-20-00327 33375828 PMC8610228

[B13] Flaherty M. M. Price R. Murgia S. Manukian E. (2024). Can playing a game improve children’s speech recognition? A preliminary study of implicit talker familiarity effects. *Am. J. Audiol.* 6 1–16. 10.1044/2023_AJA-23-00156 38056473

[B14] Garcia M. L. Cooke M. (2006). Effect of masker type on native and non-native consonant perception in noise. *J. Acoust. Soc. Am.* 119 2445–2454. 10.1121/1.2180210 16642857

[B15] Hall J. W. Grose J. H. Buss E. Dev M. B. (2002). Spondee recognition in a two-talker masker and a speech-shaped noise masker in adults and children. *Ear Hear.* 23 159–165. 10.1097/00003446-200204000-00008 11951851

[B16] Johnsrude I. S. Mackey A. Hakyemez H. Alexander E. Trang H. P. Carlyon R. P. (2013). Swinging at a cocktail party: Voice familiarity aids speech perception in the presence of a competing voice. *Psychol. Sci.* 24 1995–2004. 10.1177/0956797613482467 23985575

[B17] Karns C. M. Isbell E. Giuliano R. J. Neville H. J. (2015). Auditory attention in childhood and adolescence: An event-related potential study of spatial selective attention to one of two simultaneous stories. *Dev. Cogn. Neurosci.* 13 53–67. 10.1016/j.dcn.2015.03.001 26002721 PMC4470421

[B18] Kreitewolf J. Mathias S. R. von Kriegstein K. (2017). Implicit talker training improves comprehension of auditory speech in noise. *Front. Psychol.* 8:1584. 10.3389/fpsyg.2017.01584 28959226 PMC5603660

[B19] Leibold L. J. Buss E. (2013). Children’s identification of consonants in a speech-shaped noise or a two-talker masker. *J. Speech Lang. Hear. Res.* 56 1144–1155. 10.1044/1092-4388(2012/12-0011).Children 23785181 PMC3981452

[B20] Leibold L. J. Yarnell Bonino A. Buss E. (2016). Masked speech perception thresholds in infants, children, and adults. *Ear Hear.* 37 345–353. 10.1097/AUD.0000000000000270 26783855 PMC4844837

[B21] Levi S. V. (2015). Talker familiarity and spoken word recognition in school-age children. *J. Child Lang.* 42 843–872. 10.3109/10409238.2016.1143913.PP2A 25159173 PMC4344430

[B22] Levi S. V. Harel D. Schwartz R. G. (2019). Language ability and the familiar talker advantage: Generalizing to unfamiliar talkers is what matters. *J. Speech Lang. Hear. Res.* 62 1436–1436. 10.1044/2019_JSLHR-L-18-0160 31021674 PMC6808318

[B23] Nygaard L. C. Sommers M. S. Pisoni D. B. (1994). Speech perception as a talker-contingent process. *Psychol. Sci.* 5 42–46. 10.1111/j.1467-9280.1994.tb00612.x 21526138 PMC3081685

[B24] Pinheiro J. Bates D. DebRoy S. Sarkar D. (2016). *nlme: Linear and nonlinear mixed effects models*. R package version 3.1-125. Available online at: https://CRAN.R-project.org/package=nlme

[B25] R Core Team (2019). *R: A language and environment for statistical computing*. R Foundation for Statistical Computing. Available online at: https://www.Rproject.org/

[B26] Theodore R. M. Myers E. B. Lomibao J. A. (2015). Talker-specific influences on phonetic category structure. *J. Acoust. Soc. Am.* 138 1068–1078. 10.1121/1.4927489 26328722

[B27] Van Lancker D. Kreiman J. Emmorey K. (1985). Familiar voice recognition: Patterns and parameters Part I: Recognition of backward voices. *J. Phonet.* 13 19–38. 10.1016/s0095-4470(19)30723-5

